# Effects of feed intake restriction during late pregnancy on the function, anti-oxidation capability and acute phase protein synthesis of ovine liver

**DOI:** 10.5713/ajas.18.0331

**Published:** 2018-07-26

**Authors:** Huan Yang, Ying Wang, Chi Ma, Chuan Sun, Yingchun Liu, Kaifeng Wu, Ming Li, Gerelt Borjigin, Feng Gao

**Affiliations:** 1 College of Animal Science, Inner Mongolia Agricultural University, Hohhot 010018, China; 2 College of Life Science, Inner Mongolia Agricultural University, Hohhot 010018, China; 3 Inner Mongolia Key Laboratory of Biomanufacturing, Hohhot 010018, China; 4 College of Food Science and Engineering, Inner Mongolia Agricultural University, Hohhot 010018, China; 5 Key Laboratory of Mutton Sheep Genetics and Breeding of Ministry of Agriculture, Hohhot 010018, China

**Keywords:** Acute Phase Protein, Anti-oxidation Capability, Liver Function, Malnutrition

## Abstract

**Objective:**

An experiment was conducted to investigate the effects of feed intake restriction during late pregnancy on the function, anti-oxidation capability and acute phase protein synthesis of ovine liver.

**Methods:**

Eighteen time-mated ewes with singleton fetuses were allocated to three groups: restricted group 1 (RG1, 0.18 MJ ME/kg W^0.75^ d, n = 6), restricted group 2 (RG2, 0.33 MJ ME/kg W^0.75^ d), n = 6) and a control group (CG, *ad libitum*, 0.67 MJ ME/kg W^0.75^ d, n = 6). The feed restriction period was from 90 days to 140 days of pregnancy.

**Results:**

The ewe’s body weight, liver weights, water, and protein content of liver in the restricted groups were reduced compared with the CG group (p<0.05), but the liver fat contents in the RG1 group were higher than those of the CG group (p<0.05). The increased hepatic collagen fibers and reticular fibers were observed in the restricted groups with the reduction of energy intake. The concentrations of nonesterified free fatty acids in the RG1 and RG2 groups were higher than those of the CG group with the reduction of energy intake (p<0.05), but there were decreased concentrations of lipoprotein lipase and hepatic lipase in both restricted groups compared with the CG group (p<0.05). In addition, the increased concentrations of β-hydroxybutyric acid, triglycerides, malondialdehyde, total antioxidant capacity and activities of superoxide dismutase activity and catalase were found in the RG1 group, and the concentrations of cholinesterase in the RG1 group were reduced compared with the CG group (p<0.05). For the concentrations of acute phase proteins, the C-reactive protein (CRP) in the RG1 group were reduced compared with the CG group, but there were no differences in haptoglobin relative to the controls (p>0.05).

**Conclusion:**

The fat accumulation, increased hepatic fibrosis, antioxidant imbalance and modified synthesis of acute phase proteins were induced in ewe’s liver by maternal malnutrition during late pregnancy, which were detrimental for liver function to accommodate pregnancy.

## INTRODUCTION

Pregnancy toxemia (PT) commonly affects pregnant ewes and does during late gestation [1,2]. As a metabolic disease, PT is characterized by hypoglycemia and hyperketonemia due to a lack of ability to maintain sufficient energy balance during periods of negative energy balance and impaired gluconeogenesis [3,4]. The most common clinical signs are weakness, depression, mental dullness, disorientation, anorexia, blindness, and finally recumbency and death after 3 to 10 days [1]. Severe PT during pregnancy is the main cause of liver dysfunction and threatens the survival of the fetus and maternal [5–7]. For sheep, 40% of the affected ewes die even if treated intensively, and the offspring die before or immediately after parturition in 20% of cases [8].

The liver regulates most of the concentrations of plasma constituents, removes metabolic end products, and provides a constant source of glucose to meet energy requirements of the peripheral tissues [9]. As an essential metabolic organ [10], the liver is an important coordinating center for overall body energy homeostasis [11]. In addition, the liver plays important functions of ketone body formation and metabolism and increases synthesis of fibrinogen and other acute phase reactants in response to tissue damage [12]. More importantly, liver disorders occurring during pregnancy may be specifically pregnancy-related [13]. It appears that impaired hepatic ketone body utilization in late pregnancy facilitates development of PT [14]. Insulin resistance has also been documented to represent causative factor because impaired insulin dependent inhibition of the ketone body formation was found in ewes during late pregnancy [15]. However, the etiopathology of liver to PT is still poorly understood. Therefore, the objective of the present study was to investigate the effects of feed intake restriction during late pregnancy on the function, anti-oxidation capability and acute phase protein (APP) synthesis of ovine liver.

## MATERIALS AND METHODS

### Animal care

All experimental procedures were conducted in conformity with institutional guidelines for the care and use of laboratory animals in China (The State Science and Technology Commission of China, 1988).

### Animals and treatments

This study is a companion study, and the details of animals, experimental design and detailed procedures have been presented previously [16]. Eighteen Mongolian ewes (mean live weights 52.82±2.67 kg) in their second or third parity were mated at a synchronized oestrus. Based on the fact that the fetus is considered to achieve 80% to 85% of its final birth weight during the last two months of pregnancy [17,18], maternal undernutrition was carried out from 90 d to 140 d of gestation. At the beginning of the experiment, the 18 animals were allocated to three different groups (Table 1): restricted group1 (RG1, 0.18 MJ ME/kg W^0.75^ d, n = 6), restricted group 2 (RG2, 0.33 MJ ME/kg W^0.75^ d, n = 6), and control group (CG, *ad libitum*, 0.67 MJ ME/kg W^0.75^ d, n = 6). All animals were housed in individual pens and fed chopped hay (Table 2). Following one-week acclimatization, the amount of feed offered was constant throughout the restricted period. Restricted ewes were fed at 08:30 and 16:00 h each day. The ewes in control group were offered feed at 08:30, 11:00, and 16:00 h daily (the feed refusals were approximately 10% of the total amount offered). The animals had free access to water and mineral mixture block. The feed refusals were collected daily and recorded before feeding at 08:30 and sub-sampled for chemical analysis. At 140 d of pregnancy, six ewes in each group were slaughtered respectively, and body weight, ewe’s liver weight were measured respectively. Jugular blood samples (10 mL) in each group were collected into heparinized tubes and centrifuged (3,500 g, 15 min). The plasma samples were stored at −80°C. Some of the liver tissues were snap-frozen in liquid nitrogen and held at −80°C. Portions of the liver were fixed with paraformaldehyde (0.1 mol/L, pH 7.4), paraffin-embedded, sectioned at 4 to 6 μm and stained with commercial kits of reticulin stain (D032, NJJCBIO, Nanjing, China) and Masson stain (D026, NJJCBIO, China) for microscopic examination after fixation for at least 2 days.

### Chemical components analyses in ewe’s liver

The moisture in ewe’s liver was determined by freeze-drying to a constant weight (Christ, Alpha 1–4 lsc, German), and sample components were analyzed for chemical fat, protein and ash. The crude protein content was determined using the Kjeldahl method, and the values were converted to protein using the factor 6.25. The chemical fat content was determined as the difference in dry matter before and after extraction using ether. The ash content of the liver was the residue left after ashing at 550°C in a muffle furnace.

### CHE, LPL, HL, TG, NEFA, and BHBA in ewes

Approximately 0.5 g of the ewe’s liver was rinsed in 0.85% frozen saline. A 10% liver homogenate was used to analyze the concentrations of the acetylcholinesterase (CHE, A024), lipoprotein lipase (LPL, A067), hepatic lipase (HL, A067), in ewe’s livers by spectrophotometry (EMC-61PC-UV, Duisburg, Germany) according to the procedures of commercial kits (NJJCBIO, China). The determination of the triglyceride (TG) contents by glycerine phosphate oxidase peroxidase assay (TG, A110-2, NJJCBIO, China). The commercial kits of nonesterified free fatty acids (NEFA, A042) and β-hydroxybutyric acid (BHBA, E030-1, NJJCBIO, China) were purchased to determine the concentrations of NEFA and BHBA in maternal plasma using spectrophotometer (EMC-61PC-UV, Germany).

### T-AOC, CAT, SOD, and MDA in livers

A 10% liver homogenate was used to measure the activities of superoxide dismutase (SOD), and catalase (CAT), and the content of total antioxidant capacity (T-AOC), and malondialdehyde (MDA). The activities of SOD (A001-1) and CAT (A007), and the MDA content (A003-1) were determined spectrophotometrically using commercial kits (NJJCBIO, China) according to the procedures of Paglia and Valentine [19], Panckenko et al [20], and Placer et al [21], respectively. The T-AOC was determined using a spectrometric commercial kit (A015, NJJCBIO, China). In the reaction mixture, ferric ion was reduced by antioxidant reducing agents, and the blue complex Fe^2+^-TPTZ (2, 4, 6-tri (2- pyridyl)-s- triazine) was produced; absorbance was measured at 520 nm by spectrophotometer (723N, Shanghai, China). One unit of T-AOC was defined as the amount that increased the absorbance by 0.01 at 37°C and was expressed as units per milliliter in the tissue homogenate.

### Hp and CRP in liver

The levels of haptoglobin (Hp, H136) and C-reactive protein (CRP, H126) in the 10% ewe’s liver homogenate were analyzed by ELISA kits (NJJCBIO, China) according the instructions using microplate reader (ELX800, BIO-TEKINSTUMENTS, Winooski, VT, USA).

### Statistical analysis

All data were analyzed by using the analysis of variance procedure as implemented in SAS software [22]. Duncan’s test was used to identify significant differences between mean values. Significance was declared at p≤0.05.

## RESULTS

### Body weight, liver weights, chemical component and structure in ewe’s liver

**Figure 1:**
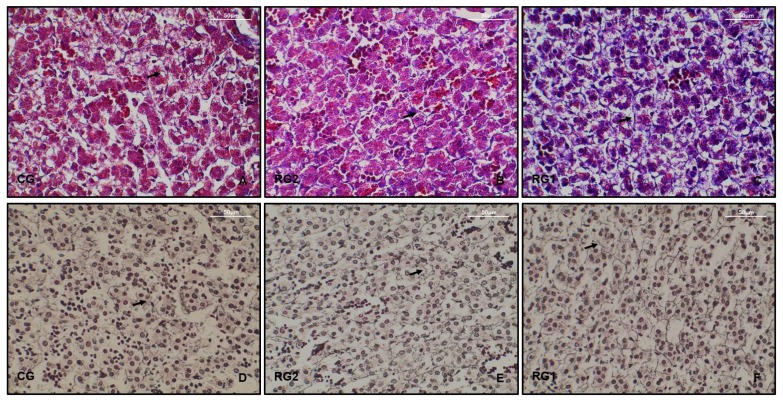
Effects of feed intake restriction during late pregnancy on collagen fiber and reticular fiber in ewes’ liver. Frames A to C from sections of ewes’ liver tissues show Masson stain for collagen fibers; Magnification, ×40 (the arrow indicates a collagen fiber). Frames D to F show reticulin stain for reticular fibers; magnification, ×40 (the arrow indicates a reticular fiber).

Effects of feed intake restriction during late pregnancy on ewe’s body weight, liver weight, chemical components and structure in ewe’s liver are presented in Table 3. The ewe’s body weight, liver weights, water and protein content of liver in the restricted groups were reduced compared with the CG group (p<0.05), but the fat contents in ewe’s liver were increased in the RG1 group (p<0.05). The water, ash and protein content expressed as percentage of ewe’s liver weight in the RG1 group were decreased compared with the CG group (p<0.05), but the ewe’s liver fat contents expressed as percentage of liver weight were higher than those of the CG group (p<0.05). In addition, the increased hepatic collagen fiber and reticular fiber in ewe’s liver were observed in both restricted groups with the reduction of ewe’s energy intake (Figure 1).

### CHE, LPL, HL, TG in ewe’s liver and NEFA, BHBA in ewe’s blood

The concentrations of CHE, LPL, HL, TG in ewe’s liver and NEFA, BHBA in blood are summarized in Table 4. The concentrations of NEFA in the RG1 and RG2 groups were higher than those of the CG group with the reduction of energy intake (p<0.05), and there were increased BHBA and TG in the RG1 group relative to the controls (p<0.05); however, the concentrations of LPL and HL in both restricted groups, and the concentrations of CHE in the RG1 group was reduced compared with the CG group (p<0.05).

### T-AOC, CAT, SOD, and MDA in ewe’s liver

Table 5 shows the effects of feed intake restriction during late pregnancy on the concentrations of T-AOC and MDA, and activities of CAT and SOD in ewes’ livers. The activities of SOD and CAT, and the T-AOC content in the RG1 group were significantly higher than those of the CG group (p<0.05); however, the concentrations of MDA in the RG1 group were increased compared with the CG group (p<0.05). For the RG2 group, there were no differences in T-AOC, SOD, glutathione peroxidase, and MDA relative to the controls (p>0.05).

### Acute phase proteins in ewe’s liver

Effects of feed intake restriction during late pregnancy on the concentrations of Hp and CRP in ewe’s liver are presented in Table 6. The concentrations of Hp in both restricted groups were increased compared with the CG group, but no differences were observed (p>0.05). For the concentration of CRP, the reduced concentration in the RG1 group was found compared with the CG group (p<0.05).

## DISCUSSION

The PT to ewe is characterized by plasma β-hydroxybutyrate concentrations usually higher than 1.6 mmol/L [3]. In this study, the increased BHBA in the RG1 group indicated that sever PT had been induced by malnutrition during late pregnancy. The severe underfeeding leads to tissue masses decreasing, and the weight loss is particularly in metabolically powerful organs, such as the liver [23,24]. Just as changes in quality and function are reflected during pregnancy, furthermore, the liver plays a pivotal role in maternal metabolism to accommodate pregnancy [25]. In this study, the reduction of ewe feed intake during late pregnancy leaded to loss of liver weight and protein content. A large amount of NEFAs, produced by body fat mobilization, entered the liver and resulted in the re-esterification of NEFAs into fats such as TG because of the decreased LPL and HL in both restricted groups. More importantly, the reduction of ewe feed intake during late pregnancy not only induced fat accumulation but the increased hepatic fibrosis in ewes. Hepatic fibrosis is a common pathological process resulting from various chronic hepatic injuries and an important cause of the disruption of normal hepatic architecture and liver dysfunction [26,27].

As a marker of the overall functional reserve of the liver, cholinesterase (CHE) synthesis is decreased markedly with hepatocyte dysfunction [28], which makes CHE a more specific indicator of liver dysfunction than traditional liver function tests [29]. In pregnancy, most liver dysfunction is pregnancy-related. In this present study, the CHE concentrations in RG1 group were reduced with the reduction of ewe’s feed intake, which is a worse signal for its liver function to accommodate pregnancy. The hepatic fibrosis might be an important cause of their liver dysfunction [30].

Oxidative stress occurs as a consequence of the imbalance between natural cellular antioxidative defenses and the prooxidant state [31]. The MDA is an index of reactive oxygen species-induced oxidative stress [32], as a metabolic product of lipid peroxides [33]. The data in this study indicated that increased MDA was produced in the RG1 group, although the increased SOD, CAT, and T-AOC were found. As an enzyme in antioxidant system, SOD promotes the conversion of an anion superoxide to H_2_O_2_ [34], and CAT is essential for all cells to eliminate H_2_O_2_ [35]. The higher concentration of MDA in the ewe’s liver suggested that oxidative stress was induced. The increased fat accumulation and worse lipid metabolism such as higher concentration of BHBA in RG1 might be important inducements for triggering oxidative stress, which would lead to liver cell damage. In addition, the liver fibrosis involves a range of causes, but most of them involve the production of free radicals [27], and oxidative stress is a key factor [36]. The oxidative stress in ewe’s liver was a cause of its hepatic fibrosis and liver dysfunction.

The acute phase response is a prominent systemic reaction of the organism to local or systemic disturbances in its homeostasis caused by the body under a variety of stressors [37,38]. In the acute phase, the APPs are usually synthesized in hepatocytes and play an important role in mediating inflammatory processes to clear cell debris and repair tissue [39], and the features of the acute phase response include increased hepatic lipogenesis and decomposition of adipose tissue [40]. An APP has been defined as one whose plasma concentration increases (positive APPs) or decreases (negative APPs) [37,40]. The positive APPs that undergo to an increase in the course of the AP response include Hp, CRP, serum amyloid A, where Hp is the primary APP in ruminants [40]. In this study, however, although the Hp had a trend to increase with the reduction of ewe feed intake, the CRP was decreased in the RG1 group. Hp possesses anti-inflammatory and immunomodulatory properties and functions as an antioxidant to prevent oxidative tissue damage [41,42]. During starvation, however, a general depression of hepatic protein synthesis occurs [37]. The hepatic oxidative stress and fibrosis that damaged the ewe’s liver cells seriously and liver dysfunction for malnourished pregnant ewe might restraint the synthesis of CRP. Pepys and Hirschfield [43] found that there is a strong positive association between CRP concentration and body mass index, and weight loss lowers the CRP value, which is in agreement with the results of our study.

In conclusion, the reduction of ewe feed intake during late pregnancy resulted in loss of liver weight and protein content, but induced fat accumulation and the increased hepatic fibrosis in ewes. The hepatic fibrosis, antioxidant imbalance, modified the synthesis of APPs and liver dysfunction were worse to accommodate pregnancy, which all might act as hepatic etiologic factor causing PT in ewes.

## Figures and Tables

**Table 1:** Planes of maternal nutrition in different groups during late pregnancy

Treatments	RG11)	RG21)	CG1) (*ad libitum*)
Mean daily hay intake (g/d)2)	440	843	1,689
Mean daily crude protein intake (g/d)	44	85	170
Daily metabolizable energy intake (MJ ME/kg W^0.75^ d)3)	0.18	0.33	0.67

ME, metabolisable energy.

1) RG1, restricted group 1; RG2, restricted group 2; CG, control group.

2) Mean daily hay intake and crude protein intake are represented on a natural basis.

3) Daily metabolizable energy intake is represented on a dry matter basis.

**Table 2:** Composition of hay and refusals during the restriction period

Items	Grass hay	Refusals
ME (MJ/kg)	8.90	-
DM (%)	88.42	91.99
CP (%)	10.09	9.27
EE (%)	4.34	2.72
NDF (%)	71.98	71.19
ADF (%)	35.82	36.60
ASH (%)	4.67	4.39
Ca (%)	0.57	0.68
P (%)	0.09	0.08

ME, metabolisable energy; DM, dry matter; CP, crude protein; EE, ether extract; NDF, neutraldetergent fiber; ADF, acid detergent fiber; Ca, calcium; P, phosphorus.

**Table 3:** Effects of feed intake restriction during late pregnancy on body weight, liver weight, chemical components and structure in ewe’s liver

Items	Day 140			SEM	p-value

	RG11)	RG2	CG		
Body weight (kg)	38.67^c^	43.85^b^	52.62^a^	1.65	0.0001
Liver weight (g)	578.25^b^	515.52^b^	682.29^a^	34.92	0.0009
Chemical component (g)					
Water (g)	321.16^b^	347.78^b^	466.59^a^	32.03	0.0009
Protein(g)	80.91^b^	103.52^b^	141.76^a^	17.71	0.012
Fat (g)	147.18^a^	38.48^b^	37.93^b^	20.84	0.0001
Ash (g)	9.03^a^	12.14^a^	13.66^a^	2.67	0.23
Chemical components expressed as percentage of liver weight (%)					
Water (%)	55.17^b^	67.46^a^	68.35^a^	3.34	0.002
Protein (%)	35.61^b^	60.87^a^	65.74^a^	6.01	0.0003
Fat (%)	55.40^a^	23.35^b^	17.86^b^	5.84	<0.001
Ash (%)	3.48^b^	7.21^a^	6.25^a^	1.21	0.016

SEM, standard error of the mean; ME, metabolisable energy.

1) RG1, restricted group 1 (0.18 MJ ME/kg W^0.75^ d); RG2, restricted group 2 (0.33 MJ ME/kg W^0.75^ d); CG, control group, *ad libitum* (0.67 MJ ME/kg W^0.75^ d).

a–c Within a row, means with different letters differ (p<0.05).

**Table 4:** Effect of feed intake restriction during late pregnancy on CHE, LPL, HL, TG in ewe’s liver and on NEFA, BHBA in ewe’s blood

Items	Day 140			SEM	p-value

	RG11)	RG21)	CG1)		
In liver					
CHE (U/mg)	13.84^b^	19.31^ab^	20.10^a^	1.25	0.021
LPL (U/mg)	2.27^b^	2.79^b^	4.03^a^	0.19	0.0003
HL (U/mg)	2.32^b^	3.05^b^	4.44^a^	0.26	0.0008
TG (mmol/g)	1.89^a^	0.51^b^	0.44^b^	0.16	0.0001
In blood					
NEFA (μmol/L)	1,236.80^a^	1,186.50^a^	415.75^b^	57.92	0.0001
BHBA (mmol/L)	2.61^a^	0.76^b^	0.53^b^	0.15	0.0001

CHE, acetylcholinesterase; LPL, lipoprotein lipase; HL, hepatic lipase; TG, triglycerides; NEFA, nonesterified free fatty acids; BHBA, β-hydroxybutyric acid; SEM, standard error of the mean.

1) RG1, restricted group 1 (0.18 MJ ME/kg W^0.75^ d); RG2, restricted group 2 (0.33 MJ ME/kg W^0.75^ d); CG, control group, ad libitum (0.67 MJ ME/kg W^0.75^ d).

a–b Within a row, means with different letters differ (p<0.05).

**Table 5:** Effect of feed intake restriction during late pregnancy on antioxidant parameters in ewes’ liver

Items	Day 140			SEM	p-value

	RG11)	RG21)	CG1)		
MDA (nmol/mL)	27.89^b^	16.67^a^	18.05^a^	2.05	0.0001
SOD (U/mL)	31.27^b^	20.71^a^	15.38^a^	3.61	0.01
CAT (U/mg)	23.58^b^	14.41^a^	12.86^a^	2.30	0.001
T-AOC (U/mL)	5.17^b^	3.55^ab^	2.94^a^	0.76	0.031

SEM, standard error of the mean; MDA, malondialdehyde; SOD, superoxide dismutase; CAT, catalase; T-AOC, total antioxidant capacity.

1) RG1, restricted group 1 (0.18 MJ ME/kg W^0.75^ d); RG2, restricted group 2 (0.33 MJ ME/kg W^0.75^ d); CG, control group, ad libitum (0.67 MJ ME/kg W0.75 d).

a–b Within a row, means with different letters differ (p<0.05).

**Table 6:** Effect of feed intake restriction during late pregnancy on acute phase protein in ewes’ liver

Items	Day 140			SEM	p-value

	RG11)	RG21)	CG1)		
Hp (μg/mL)	17.81^a^	12.56^a^	10.59^a^	4.32	0.24
CRP (μg/mL)	93.52^b^	154.58^ab^	181.80^a^	36.86	0.01

SEM, standard error of the mean; Hp, haptoglobin; CRP, C-reactive protein.

1) RG1, restricted group 1 (0.18 MJ ME/kg W^0.75^ d); RG2, restricted group 2 (0.33 MJ ME/kg W^0.75^ d); CG, control group, ad libitum (0.67 MJ ME/kg W^0.75^ d).

a–b Within a row, means with different letters differ (p<0.05).
